# Advanced practice nursing in Brazil: bibliometric analysis of dissertations and theses

**DOI:** 10.1590/1980-220X-REEUSP-2024-0253en

**Published:** 2025-01-13

**Authors:** Ana Clara Dantas, Mércio Gabriel de Araújo, Jéssica Naiara de Medeiros Araújo, Ana Beatriz Marinho de Medeiros, Pedro Henrique Azevedo dos Santos, Bárbara Ebilizarda Coutinho Borges, Jéssica Dantas de Sá Tinôco, Héllyda de Souza Bezerra

**Affiliations:** 1 Universidade Federal do Rio Grande do Norte, Natal, RN, Brazil.; 2 Universidade Federal do Rio Grande do Norte, Santa Cruz, RN, Brazil.; 3 Universidade do Estado do Rio Grande do Norte, Caicó, RN, Brazil.

**Keywords:** Advanced Practice Nursing, Nursing Research, Health Postgraduate Programs, Scientific Production Indicators, Bibliometrics, Enfermería de Práctica Avanzada, Investigación en Enfermería, Programas de Posgrado en Salud, Indicadores de Producción Científica, Bibliometría

## Abstract

**Objective:**

To analyze the scientific production on Advanced Practice Nursing based on dissertations and theses published in Brazil.

**Method:**

A bibliometric study, with a descriptive approach based on documents, carried out in the Coordination for the Improvement of Higher Education Personnel Theses and Dissertations Bank, Scientific Electronic Library Online, Virtual Health Library, Latin American and Caribbean Literature in Health Sciences and institutional repositories, from October to November 2023.

**Results:**

Of the 25 scientific productions identified, 16 are dissertations and nine are theses. Most were published in 2022 (40.0%), in the Southeast region (60.0%), especially at the *Faculdade Israelita de Ciências da Saúde Albert Einstein*, through a professional master’s program in nursing (20.0%). Family health was the most evident area of specialty in the sample (36.0%), and the prevalent level of complexity was Primary Health Care (76.0%).

**Conclusion:**

The scenario of scientific productions on the subject in the country has begun to advance in recent years, but is still incipient. The mapping of theses and dissertations on Advanced Practice Nursing points to the emerging need to meet the demands arising from the health sector at an international and national level.

## RESUMEN

**Objetivo:**

Analizar la producción científica sobre Enfermería de Práctica Avanzada a partir de disertaciones y tesis publicadas en Brasil.

**Método:**

Estudio bibliométrico, con enfoque descriptivo basado en documentos, realizado en el Banco de Tesis y Disertaciones de la Coordinación para el Perfeccionamiento del Personal de Educación Superior, Biblioteca Científica Electrónica en Línea, Biblioteca Virtual en Salud, Literatura Latinoamericana y del Caribe en Ciencias de la Salud. y repositorios institucionales, de octubre a noviembre de 2023.

**Resultados:**

De las 25 producciones científicas identificadas, 16 son disertaciones y nueve tesis. La mayoría fueron publicadas en 2022 (40,0%), en la región Sudeste (60,0%), especialmente en la Faculdade Israelita de Ciências da Saúde Albert Einstein, a través de la maestría profesional en enfermería (20,0%). La salud de la familia fue el área de especialidad más destacada en la muestra (36,0%), y el nivel de complejidad prevalente fue la Atención Primaria de Salud (76,0%).

**Conclusión:**

El escenario de la producción científica sobre el tema en el país ha comenzado a avanzar en los últimos años, pero aún está en pañales. El mapeo de tesis y disertaciones sobre Enfermería de Práctica Avanzada apunta a la necesidad emergente de atender las demandas derivadas del sector salud a nivel internacional y nacional.

## INTRODUCTION

The World Health Organization (WHO) is constantly discussing measures to meet health demands, overcoming challenges such as the shortage of human resources and disparities in access to health. Taking into account populations’ health needs, it is essential to have health models with the potential to innovate and improve health services(1). In countries such as Canada and the United States, Advanced Practice Nursing (APN) plays a relevant role in driving innovations in health systems, improving access to health services and achieving adequate health outcomes while contributing to reducing costs(2,3).

According to data from the State of the World’s Nursing 2020 report, the existence of APN is 55% among countries in the Americas region, being more frequent in countries with a lower density of medical professionals, which contributes to expanding access to primary health care, especially in rural areas and for populations in vulnerable situations(1). In Brazil, regional disparities are evident, with better health indicators in the southeast and south and worse in the north and northeast. In this scenario, APN can be a strategy to be considered to expand access and improve the resolution of health services, aligned with Brazilian Health System (In Portuguese, *Sistema Único de Saúde* - SUS) principles and guidelines, supported by legislative frameworks, such as the Brazilian National Primary Care Policy, Professional Practice Law, regulations issued by the professional council, in addition to emphasis on evidence-based practice(4).

According to the International Council of Nurses (ICN), APN involves advanced nursing interventions that directly impact health outcomes of individuals, families, and communities. APN is based on graduate training and requires the definition of specific criteria for certification, registration, and development of essential competencies for practice(3). A conceptual analysis defined APN as “a generalist or specialised nurse who has acquired, through additional graduate education (minimum of a master’s degree), the expert knowledge base, complex decision-making skills and clinical competencies for Advanced Nursing Practice, the characteristics of which are shaped by the context in which they are credentialed to practice”(5).

The Pan American Health Organization (PAHO)/WHO defines advanced practice nurses (APNs) as a professional with graduate training who, working in an integrated manner in interprofessional teams, contributes to managing the care of patients with mild acute diseases and diagnosed chronic conditions, in accordance with clinical guidelines or protocols(6).

Despite technological advances in health, scholars point out that health systems are not prepared to manage the health demands of the coming years. WHO data points to a global deficit of 5.9 million nurses, with the largest gaps found in countries in Africa, Southeast Asia and some parts of Latin America(1). In early 2023, the ICN published the report “Recover to rebuild: investing in the nursing workforce for health system effectiveness”, in which it points out that the shortage of nurses must be addressed as a global health emergency(7).

In addition to training new nurses, it is essential to invest in advanced practice training for nurses who are already working(8). However, APN implementation is still far from becoming a reality in Brazil. In order to advance this implementation, it is crucial to provide nurses with opportunities for professional development, promote access to formal nursing education at the graduate level, implement policies that improve career and job market prospects, and consider actions to raise awareness of APN on the part of civil society, with the involvement of the government, the educational sector and regulatory institutions being essential(9). Reinforcing the relevance of the topic in Brazil today, the Federal Nursing Council (In Portuguese, *Conselho Federal de Enfermagem* - COFEN) released on June 7, 2023 Technical Note 001/2023, which addresses APN concepts, actions, implementation and regulation in Brazil(10).

In this regard, the analysis of scientific production in APN, based on theses and dissertations published by nurses in Brazil, is justified by the need to understand the current state and trends in this field of activity. Moreover, this analysis sparks reflection on the construction of knowledge and nursing practice focused on the topic, which can direct future efforts, with a view to strengthening the theoretical and practical basis of APN in Brazil.

Considering the explanations presented, the research question is: what is the overview of scientific production of dissertations and theses published about APN in Brazil? Thus, this study aims to analyze the scientific production on APN based on dissertations and theses published in Brazil.

## METHOD

### Study Design

This is a bibliometric study, with a descriptive approach based on documents. Bibliometrics is a research approach that involves the quantification of scientific production and communication, with the purpose of disseminating works, publishing publications, assessing the productivity of authors and institutions, revealing the development of science and the impact of publications in the context studied(11).

Thus, this study is configured as bibliographic research, using previously published materials on APNs. According to Marconi and Lakatos (2017), bibliographic research is not limited to reproducing what has already been published, but seeks to offer an analysis from a new perspective, with the aim of reaching innovative conclusions and guiding future actions(12).

### Study Protocol

A protocol was previously structured to guide the study development. The research report was guided by the Preferred Reporting Items for Bibliometric Analysis (PRIBA), which is an instrument used to assess items required for bibliometric studies(13).

### Data Collection

The descriptor “Advanced Practice Nursing”, indexed in the Health Sciences Descriptors (In Portuguese, *Descritores em Ciências da Saúde* - DeCS), was selected as the main focus of the research, as it is the object of study. It is worth mentioning that a pilot test was carried out, using the selected descriptor, to visualize how the research appeared in each search engine and to identify possible keywords on the topic.

After this process, keywords in Portuguese, such as “*Prática Avançada em Enfermagem*”, “*Enfermagem de Prática Avançada*” and “*Enfermagem em Prática Avançada*”, were observed in the studies. Thus, in addition to the indexed descriptor, it was decided to consider the identified keywords in order to expand the search for scientific productions.

To select the studies, a search was carried out by two researchers between October and November 2023, through the Coordination for the Improvement of Higher Education Personnel (In Portuguese, *Coordenação de Aperfeiçoamento de Pessoal de Nível Superior* - CAPES) Theses and Dissertations Bank and in the Scientific Electronic Library Online (SciELO), Virtual Health Library (VHL) and Latin American and Caribbean Literature in Health Sciences (LILACS) databases, taking into account their particularities, with the following strategy: “*Prática Avançada de Enfermagem*” OR “*Prática Avançada em Enfermagem*” OR “*Enfermagem de Prática Avançada*” OR “*Enfermagem em Prática Avançada*”.

Institutional repositories of Brazilian graduate programs were also consulted. In then, the descriptor and keywords were used individually without the use of Boolean operators. Institutional repositories were identified by accessing the *Sucupira* Platform, in the Evaluated and Recognized Courses tab, with the “Nursing” filter in the Evaluation Area item(14). In this way, it was possible to issue a list of 61 higher education institutions (HEIs) with 85 recognized graduate programs in nursing in Brazil.

### Selection Criteria

Studies that addressed the topic and were classified as theses or dissertations and published in Brazil were included. No time frame was established in order to broaden study selection. Duplicate studies were removed from the sample.

### Data Analysis and Treatment

An instrument was developed to extract the information contained in productions, with variables related to identification data such as: title, author, advisor, co-advisor, degree, type of production, year of publication, institution, graduate program and graduate program concentration area, financing (code) and funding agency; methodological aspects, including objective, research question, theoretical and/or methodological and/or philosophical framework, methodology, area of specialty (specific field of knowledge of nursing addressed in the research) and level of health care (primary, secondary or tertiary level, referring to the level of complexity of health services); and main findings, with results and/or impressions of studies and limitations found.

In the variables related to the findings (results and/or impressions of studies and limitations found), thematic analysis was performed with an inductive approach and based on compiled data. After collecting the data, an exhaustive and detailed reading was carried out, allowing a complete immersion in the content of studies. The data were grouped to form potential topics that were later refined to capture the nuances present in the texts analyzed(15). All variables were analyzed using simple descriptive statistics, recording absolute and relative frequencies.

### Ethical Aspects

Since the research used secondary data in the public domain available in the literature, approval by a Research Ethics Committee (REC) was not required. However, it is worth noting that the copyright of the studies analyzed was properly respected.

### Data Availability

The dataset for this study was made available in the public repository Harvard Dataverse(16).

https://doi.org/10.7910/DVN/1N7KSG

## RESULTS

**Figure 1:**
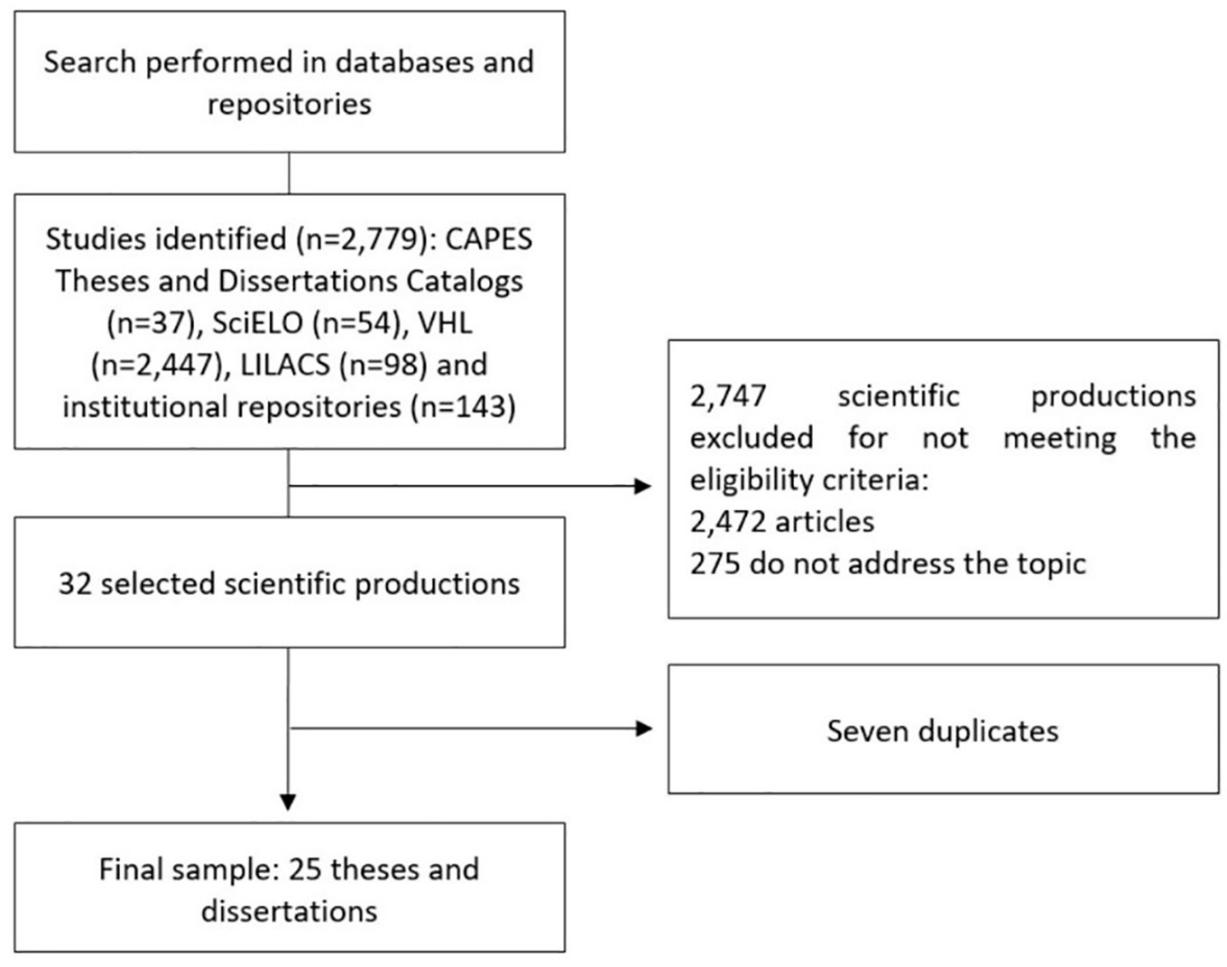
Sample search and selection process flowchart. Natal, RN, Brazil, 2023.

A total of 2,779 scientific productions were identified, of which 25 remained in the sample. Of these, 16 are dissertations and nine are theses (Figure 1).

Table 1 presents he characterization of the studies analyzed according to year of publication, geographic region of the country, educational institution, graduate program concentration area, theoretical and/or methodological and/or philosophical framework used, study design, type of approach, area of specialty and level of health care.

**Table 1:** Characterization of scientific productions of theses and dissertations – Natal, RN, Brazil, 2023.

Variable	N (%)
**Year of publication**	
2023	05 (20.0)
2022	10 (40.0)
2021	05 (20.0)
2020	03 (12.0)
2019	01 (4.0)
2018	01 (4.0)
**Geographic region**	
Southeast	15 (60.0)
South	05 (20.0)
Northeast	03 (12.0)
Midwest	02 (8.0)
**Educational institution**	
*Faculdade Israelita de Ciências da Saúde Albert Einstein*	05 (20.0)
*Universidade de São Paulo*	04 (16.0)
*Universidade Federal do Paraná*	02 (8.0)
*Universidade Federal do Rio Grande do Norte*	02 (8.0)
*Universidade Federal do Estado do Rio de Janeiro*	02 (8.0)
*Instituto Sírio-Libanês de Ensino e Pesquisa*	01 (4.0)
*Escola Nacional de Saúde Pública Sérgio Arouca da Fundação Oswaldo Cruz*	01 (4.0)
*Universidade Estadual de Campinas*	01 (4.0)
*Escola de Enfermagem Aurora de Afonso Costa, Universidade Federal Fluminense*	01 (4.0)
*Universidade Estadual do Oeste do Paraná*	01 (4.0)
*Universidade Federal de Santa Catarina*	01 (4.0)
*Universidade Federal de Ciências da Saúde de Porto Alegre*	01 (4.0)
*Universidade Federal de Sergipe*	01 (4.0)
*Universidade de Brasília*	01 (4.0)
*Universidade Católica de Brasília*	01 (4.0)
**Graduate program concentration area**	
Professional practices in health and nursing	05 (20.0)
Nursing in public health	03 (8.0)
Professional practices in nursing	02 (8.0)
Nursing in health care	02 (8.0)
Technology and innovation in nursing	01 (4.0)
Care and technological innovation in health and nursing	01 (4.0)
Teaching in health and its interfaces with the SUS	01 (4.0)
Nursing, health and care in society	01 (4.0)
Health practices and policies	01 (4.0)
Management and care in the context of the SUS and policies in health and nursing	01 (4.0)
Policies, planning, management and care in health	01 (4.0)
Education and work in health and nursing	01 (4.0)
Theoretical, methodological and technological foundations of the care process	01 (4.0)
Fundamental, cultural, environmental and historical bases of health care	01 (4.0)
Longevity and quality of life	01 (4.0)
Integration of teaching and health services in professional training	01 (4.0)
Applied research in oncology	01 (4.0)
**Theoretical and/or methodological and/or philosophical framework**	
*Theoretical*	
ICN Advanced Practice Nursing	06 (24.0)
Nursing Process	04 (16.0)
Zarifian Competencies	03 (12.0)
Horkheimer’s Critical Theory	01 (4.0)
Edgar Morin’s Complexity Theory	01 (4.0)
Neo-Institutionalism	01 (4.0)
*Methodological*	
Implementation Science	01 (4.0)
*Philosophical*	
Karl Marx’s Historical-Dialectical Materialism	01 (4.0)
*They did not describe any reference of any nature*	06 (24.0)
**Study design**	
Exploratory study	10 (40.0)
Methodological study	06 (24.0)
Cross-sectional study	02 (8.0)
Implementation study	02 (8.0)
Mixed methods study	02 (8.0)
Descriptive study	01 (4.0)
Reflective study	01 (4.0)
Convergent care research	01 (4.0)
**Type of approach**	
Mixed	14 (56.0)
Quantitative	06 (24.0)
Qualitative	05 (20.0)
**Area of specialty**	
Family health	09 (36.0)
Oncology nursing	03 (12.0)
Nursing process	02 (8.0)
Child health	02 (8.0)
Elderly health	02 (8.0)
Public health	01 (4.0)
Emergency care nursing	01 (4.0)
Management of health and nursing actions and services	01 (4.0)
Intensive care	01 (4.0)
Obstetric nursing	01 (4.0)
Women’s health	01 (4.0)
Mental health	01 (4.0)
**Level of health care**	
Primary level	19 (76.0)
Tertiary level	04 (16.0)
Secondary level	02 (8.0)

The results and/or impressions of studies were analyzed and demonstrated potential and barriers to implementing APN, as presented in Table 2.

**Table 2:** Potentials and barriers to implementing Advanced Practice Nursing – Natal, RN, Brazil, 2023.

Potential for implementing Advanced Practice Nursing	N (%)
Training for Advanced Practice Nursing	22 (44.0)
Public policies for Primary Health Care	08 (32.0)
Nursing Professional Practice Law	08 (32.0)
Practices currently being carried out that are considered as Advanced Practice Nursing	04 (16.0)
**Barriers to implementing Advanced Practice Nursing**	
Need for reformulation of current legislation	03 (12.0)
Professional corporatism	03 (12.0)
Lack of clarity in the professional role in Advanced Practice Nursing	03 (12.0)
Lack of care management skills	02 (8.0)
Shortage of specific teaching staff for training in Advanced Practice Nursing	01 (4.0)

The limitations of studies were also analyzed, and it was observed that most were related to the methodological limitations of the chosen study design (44.0%). The COVID-19 pandemic scenario, linked to the overload of nurses during this period, was also characterized as a limiting factor (16.0%), as well as the lack of specific legislation in Brazil for the exercise of APN (8.0%) and social and cultural limitations described during a sandwich doctoral program abroad (4.0%). In five productions (20.0%), no limitations were described.

The general statement of scientific productions was arranged according to region, title, author, year, type of production, institution, financing (code) and funding agency (Chart 1).

**Chart 1:** General statement of selected scientific productions – Natal, RN, Brazil, 2023.

Title	Author (year)	Type of production	Institution	Financing (code)	Funding agency
**Southeast**					
*Consulta de enfermagem em saúde da criança e competências para enfermeiros de prática avançada*	Keila Gisele Lima Reis (2023)	Master’s dissertation – professional	*Faculdade Israelita de Ciências da Saúde Albert Einstein*	Yes (001)	CAPES/COFEN
*Consulta de enfermagem à pessoa idosa na atenção primária à* *Saúde: análise das competências propostas para enfermeiro de prática avançada*	Nayara Vilela de Farias Serranegra (2023)	Master’s dissertation – professional	*Faculdade Israelita de Ciências da Saúde Albert Einstein*	Not reported	Not reported
*Consultas de enfermagem em saúde mental na atenção primária: análise das competências para enfermeiro de prática avançada*	Patrícia Aline de Almeida (2023)	Master’s dissertation – professional	*Faculdade Israelita de Ciências da Saúde Albert Einstein*	Yes (001)	CAPES/COFEN
*Eventos agudos na Atenção Primária à Saúde: uma análise das competências de prática avançada em consultas de Enfermagem*	Marília Orlandelli Carrer (2023)	Master’s dissertation – professional	*Faculdade Israelita de Ciências da Saúde Albert Einstein*	Yes (001)	CAPES/COFEN
*Competências de prática avançada nas consultas de enfermagem em saúde da mulher na atenção primária: estudo multicêntrico*	Carla Pereira Barreto (2023)	Master’s dissertation – professional	*Faculdade Israelita de Ciências da Saúde Albert Einstein*	Yes (001)	CAPES/COFEN
*Desenvolvimento do modelo de atuação da enfermagem de prática avançada no cuidado do paciente com câncer colorretal*	Michelle Artioli Domingues (2022)	Doctoral thesis – academic	*Instituto Sírio-Libanês de Ensino e Pesquisa*	Not reported	Not reported
*Processo e relações de trabalho das Enfermeiras na Atenção Primária à Saúde: uma abordagem institucional a partir do modelo de gestão do município do Rio de Janeiro/RJ*	Priscilla Oliveira da Silva (2022)	Doctoral thesis – academic	*Escola Nacional de Saúde Pública Sérgio Arouca da Fundação Oswaldo Cruz*	Yes (001)	CAPES
*Adaptação Cultural e Validação do Inventario para la Evaluacion de Competencias En Enfermeras de Practica Avanzada (IECEPA) para a cultura Brasileira*	Flávia Carvalho Pena Dias (2022)	Master’s dissertation – academic	*Universidade Estadual de Campinas*	Yes (001)	CAPES
*Consulta de enfermagem de 1ª vez em quimioterapia: contribuições para a prática avançada em oncologia no atendimento ambulatorial*	Rubislene Assis Santos de Brito (2022)	Master’s dissertation – academic	*Universidade Federal do Estado do Rio de Janeiro*	Not reported	Not reported
*Prática Avançada de Enfermagem na Estratégia Saúde da Família: necessidade de aplicativo para a comunicação entre enfermeiro e usuário*	Emerson Willian Santos de Almeida (2022)	Master’s dissertation – academic	*Escola de Enfermagem de Ribeirão Preto, Universidade de São Paulo*	Yes (001)	CAPES
*Redução da contenção mecânica em Unidade de Terapia Intensiva: implementação de um programa de melhoria*	Bruna Luísa Melo de Aquino Lemos Corrêa (2022)	Master’s dissertation – professional	*Escola de Enfermagem Aurora de Afonso Costa, Universidade Federal Fluminense*	Yes (001)	CAPES
*A Enfermagem de Prática Avançada no processo de trabalho do enfermeiro da Atenção Primária à Saúde no município do Rio de Janeiro*	Antonio da Silva Ribeiro (2021)	Doctoral thesis – academic	*Universidade Federal do Estado do Rio de Janeiro*	Not reported	Not reported
*Vivências dos enfermeiros em práticas avançadas nos serviços de atendimento móvel de urgências*	Giane Alves Stefani (2020)	Master’s dissertation – professional	*Escola de Enfermagem de Ribeirão Preto, Universidade de São Paulo*	Not reported	Not reported
*Práticas avançadas de enfermagem na atenção primária à saúde: subsídios para o desenvolvimento e a implementação em um sistema local de saúde*	Manoel Vieira de Miranda Neto (2019)	Doctoral thesis – academic	*Universidade de São Paulo* School of Nursing	Not reported	Not reported
*Competências para práticas avançadas de enfermagem na atenção primária à saúde no contexto brasileiro*	Talita Rewa (2018)	Master’s dissertation – academic	*Universidade de São Paulo* School of Nursing	Not reported	Not reported
**South**					
*Marcadores da prática avançada de enfermagem em saúde da criança na atenção primária à saúde*	Ana Paula Dezoti (2022)	Doctoral thesis – academic	*Universidade Federal do Paraná*	Not reported	Not reported
*Validação e adaptação transcultural do instrumento* Modified Advanced practice nursing role delineation tool *para o Português do Brasil*	Kamila Caroline Minosso (2022)	Master’s dissertation – academic	*Universidade Estadual do Oeste do Paraná*	Not reported	Not reported
*Enfermagem de prática avançada em oncologia: proposta de formação profissional*	Franciane Schneider (2021)	Doctoral thesis – academic	*Universidade Federal de Santa Catarina*	Yes (001)	CAPES
*Competências do enfermeiro em práticas avançadas de Enfermagem na atenção primária à saúde*	Luis Fernando Gualdezi (2021)	Master’s dissertation – academic	*Universidade Federal do Paraná*	Yes (001)	CAPES
*Enfermagem de Práticas Avançadas: Matriz Temática para o Desenvolvimento de Protocolos na Atenção Primária em Saúde*	Ernanda Mezaroba (2020)	Master’s dissertation – professional	*Universidade Federal de Ciências da Saúde de Porto Alegre*	Not reported	Not reported
**Northeast**					
*Práticas de enfermagem no contexto da Atenção Primária à Saúde (APS) em Sergipe*	Yandra Dirce Nascimento de Castro (2022)	Master’s dissertation – academic	*Universidade Federal de Sergipe*	Not reported	Not reported
*Prática avançada de enfermagem: reflexões para subsidiar a implementação na atenção primária a saúde brasileira*	Marjorie Dantas Medeiros Melo (2021)	Doctoral thesis – academic	*Universidade Federal do Rio Grande do Norte*	Yes (001)	CAPES
*Prática avançada de enfermagem norte-americana: subsídios para reflexões da implementação no contexto obstétrico brasileiro*	Isadora Costa Andriola (2020)	Doctoral thesis – academic	*Universidade Federal do Rio Grande do Norte*	Yes (001)	CAPES
**Midwest**					
*Prática de enfermagem na Atenção Primária à Saúde no estado da Paraíba: teoria, crítica, abordagens e correlações com a* Advance Nurse Practice *(ANP)*	José da Paz Oliveira Alvarenga (2022)	Doctoral thesis – academic	*Universidade de Brasília*	Yes (not specified)	COFEN
*Grau de satisfação dos usuários idosos com o modelo internacional de atuação do enfermeiro*	Isis Michelle Pereira de Castro (2021)	Master’s dissertation – academic	*Universidade Católica de Brasília*	Not reported	Not reported

**Figure 2:**
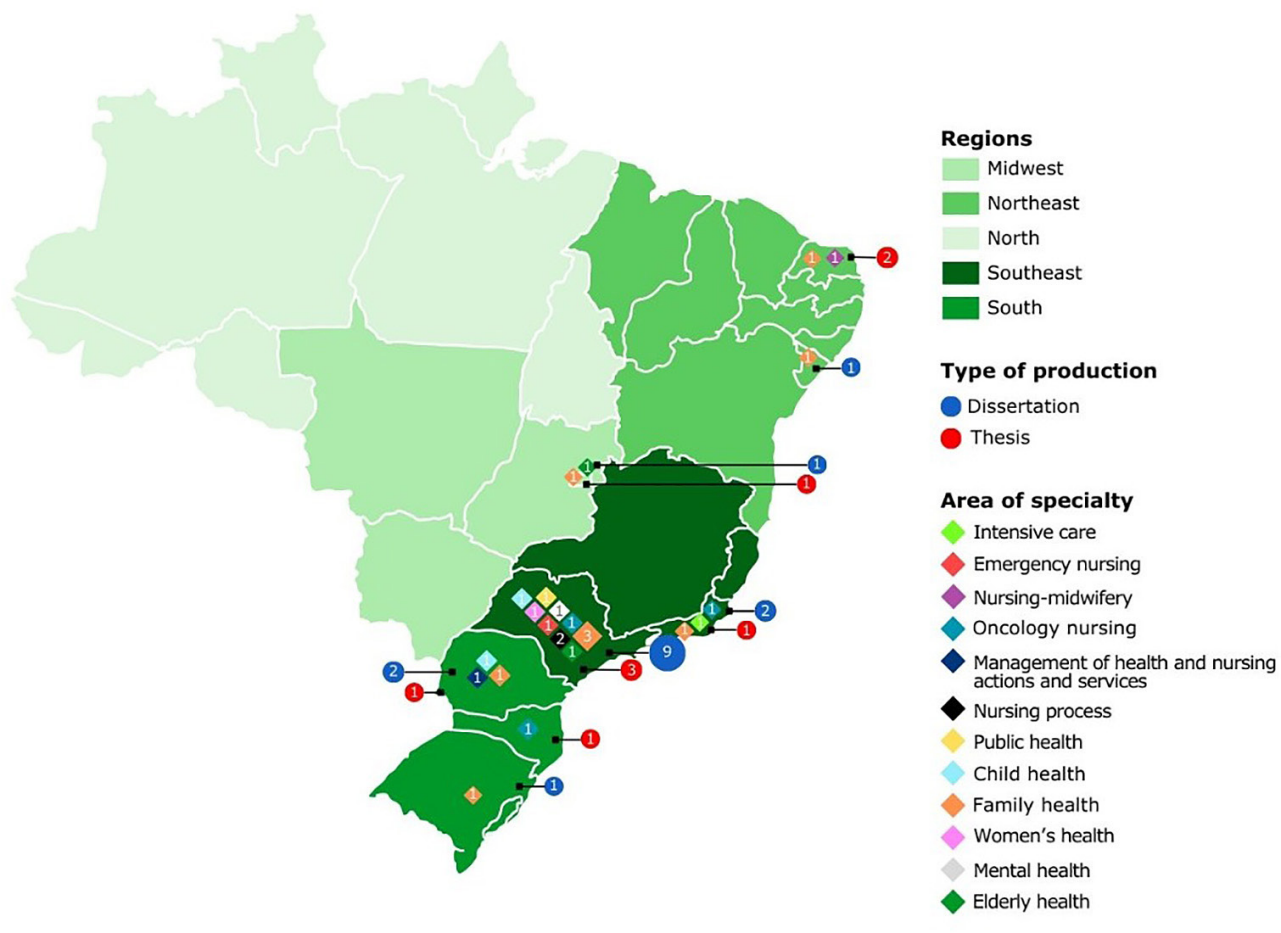
Distribution of scientific productions according to Brazilian region, type of production and area of specialty. Natal, RN, Brazil, 2023.

Figure 2 shows the distribution of scientific productions according to the Brazilian region, type of production and area of specialty.

## DISCUSSION

Most scientific productions were developed by masters, with an emphasis on academic training. This finding may be associated with the number of masters and doctoral courses. In the current scenario in Brazil, there is a greater number of masters programs compared to doctoral programs, and courses focused on academic training are more evident than those focused on professional training(17).

All productions were identified as having been developed in the last five years. Since 2013, PAHO/WHO has been driving APNs initiative by disseminating documents to address the limited human resources for universal access to health. PAHO/WHO has encouraged Latin American and Caribbean countries to develop and implement APNs in health systems, especially in PHC(18). In 2018, PAHO/WHO launched the document “Expanding the roles of nurses in primary health care”, highlighting the importance of nursing in improving access to health services, highlighting the strategies needed for APN development, implementation and assessment(6).

APN was initially discussed by national bodies in 2015. Under the presidency of Dr. Angela Maria Alvarez (2013–2016), the Brazilian Nursing Association (In Portuguese, *Associação Brasileira de Enfermagem* - ABEn) actively participated in discussions on APN in Latin America at events in the United States and Canada. Moreover, ABEn presented contributions at the XII Conference on Nursing Education on “Advanced Practices in Nursing in the Ibero-American Region”, and coordinated a working group with CAPES to develop guidelines for APN in PHC in Brazil(18).

In 2015, ABEn and COFEN participated in a meeting at PAHO headquarters to review the concept of APNs, discuss its proposal in Brazil, and assess the contributions of nursing in PHC within the scope of public policies. This effort resulted in the document “Advanced practice nursing training in primary health care: a proposal for Brazil,” which suggested expanding the scope of nursing focused on PHC and strengthening the SUS(18). In 2016, COFEN established the Commission for Advanced Practices in Nursing, with the aim of expanding the professional scope and fostering multidisciplinary collaboration in PHC(19). From this, they committed to developing scientific productions on APNs.

There was also an increase in studies on the subject published in national journals in recent years, which supports the increase in scientific productions in the same period identified in our study^(20–22)^.

The findings of our study indicate that the Southeast region has the highest number of productions on APNs. In contrast, no production was identified in the North region. It is believed that this finding is related to the discrepant number of graduate programs between the regions of the country. The North region has only four programs (two academic master’s degrees and two professional master’s degrees), a number considerably lower than that of the Southeast (30 programs), Northeast (21 programs), South (17 programs) and Midwest (seven programs) regions(14). This result suggests the need for incentives that enable this region to contribute to the advancement of science, through the establishment of new programs and the development of research from a regional point of view, since Brazil is a country characterized by high cultural and health diversity, and each region has particularities and specificities, thus considering the relevance of the North region participation(23).

Most of productions were developed at the *Faculdade Israelita de Ciências da Saúde Albert Einstein*. This highlight may be related to the program’s concentration area, “professional practices in health and nursing”, to the funding linked to the research and to the advisor. All of the institution’s research received support from Notice 28/2019 - CAPES/COFEN, which granted funding for professional master’s courses in nursing at HEIs. The advisor responsible for all the work, Dr. Manoel Vieira de Miranda Neto, has as his main research areas PHC, APNs, human resources in health and interprofessional education(24).

The exploratory study was the most considered methodology for developing the productions. It is believed that it is related to the objectives of the researches that, in their majority, sought to identify nursing professionals’ competencies in APN. Studies point out the urgency of identifying these competencies in the Brazilian context in order to achieve advanced practice implementation(10,25).

When we consider the level of health care in which the research was developed, PHC was the most common level in the sample. Consequently, the specialty area with the highest number of productions was family health. This result is in line with other research, since nursing actions in PHC in Brazil are more similar to advanced practices(20). Study addresses an effective model to support the process of developing and implementing APN in PHC, indicating possible contributions to strengthening nursing as a professional category and its leading role in the national health system(26).

Among the potential for APN in the Brazilian context, the proposal that the association of professional residency in health with a professional master’s degree represents a promising possibility for APN training stands out. In 2021, CAPES formed a working group to discuss APN training in Brazil. In 2022, five proposals for APN training were submitted to CAPES. However, in 2023, the result indicated that none of the proposals were approved, with the argument that APN is not regulated in Brazil. Researchers in the field disagree with this decision, arguing that the lack of regulation, which is under discussion, should not prevent the creation of new training processes(27,28).

Several elements can contribute to defining the professional scope and regulation, including Professional Practice Law, the Brazilian National Primary Care Policy and the lines of care recognized by the Ministry of Health. Within the scope of PHC, APN has the potential to go beyond the biomedical, curative and interventionist model, by incorporating a more comprehensive and integrative approach, such as the expanded clinic(29).

There are already activities considered as APNs in the Brazilian scenario, including risk stratification with advanced health assessments, regulation with referral and counter-referral, decision-making on prescribing medications and treatments, and requesting laboratory tests and imaging tests such as ultrasound(10,30). Some advanced practice activities, such as intrauterine device insertion, can also be performed by nurses, although municipal protocols are required(31).

Regarding the barriers to APN in the Brazilian context, the studies highlight the need to reformulate the current legislation in order to ensure professional autonomy in decision-making. There are concerns about how the country will develop the training of nurses in this practice, especially considering the shortage of specific teaching staff. In addition to this, barriers such as health professions’ corporatism and lack of clarity in the role of nursing professionals in APN were identified in advanced practice implementation. The studies emphasize the importance of carrying out collaborative studies involving regulatory bodies, managers, coordinators, professionals and users in order to clearly define the profile of APN(32,33).

Concerning the limitations identified in the studies, methodological limitations are considered expected, since every scientific study design has its inherent limitations. However, it is important to note that the context of the COVID-19 pandemic and the overload faced by nurses during this period may have impacted both the conduct of research and the progress in implementing specific legislation for APN(34,35).

It is understood that APN in Brazil is in the process of implementation due to the need for further studies on the Brazilian context, the regulation of educational and assistance policies for training nurses with APN skills, and the need for ethical support aligned with the Brazilian health system(22). Based on the results of our study, we suggest encouraging research focused on APNs, especially in the North, Midwest, and Northeast regions of Brazil. This may involve financial support for research projects, curricular changes, and/or implementation of graduate programs with an emphasis on APN training.

Limitations of this study include the possible concealment of productions that, despite efforts to identify them in various databases and institutional repositories, may not have been identified. Furthermore, it was not possible to assess the quality of materials in terms of internal consistency and to perform bibliographic coupling analysis to identify the sharing of common or similar references in the content of materials.

This study contributes to the advancement of nursing knowledge by presenting trends in scientific production about APN in Brazil. Gaps in the production of studies were identified in some regions that could offer a significant contribution to the discussion of APN in the country. It is noteworthy that filling these gaps requires greater investment and attention dedicated to the process of encouraging research in this specific field.

It also contributes by listing the areas of specialty and the different levels of health care in which production on APN has been conducted in Brazil. This indicates the areas that have potential for advanced practice implementation, in addition to suggesting contexts that are conducive to future research. Additionally, the findings indicated that research from graduate studies is still limited, especially considering the possibilities of covering content and methodological approaches that can contribute to scientific production on APN. Therefore, it is recommended that new studies be carried out to fill these gaps.

## CONCLUSION

It is concluded that most of scientific productions of theses and dissertations on APN in Brazil were published in 2022, mainly in the Southeast region, with emphasis on the *Faculdade Israelita de Ciências da Saúde Albert Einstein*. These productions focused especially on family health, within the context of PHC. The scenario of theses and dissertations on APN in the country has begun to advance in recent years, but it is still incipient, considering the potential that can be explored through additional studies. The mapping of theses and dissertations on APN points to the emerging need to meet the demands arising from the health sector at international and national levels.
