# Supplementary material for: Advanced practice nursing in Brazil: bibliometric analysis of dissertations and theses
Source: Rev Esc Enferm USP. 2025 Jan 13;58:e20240253. doi: 10.1590/1980-220X-REEUSP-2024-0253en (PMC11740733; doi:10.1590/1980-220X-REEUSP-2024-0253en)
Supplement: Translation

# Translation: Prática avançada de enfermagem no Brasil: análise bibliométrica de dissertações e teses

**DOI:** 10.1590/1980-220X-REEUSP-2024-0253pt

**Authors:** Ana Clara Dantas, Mércio Gabriel de Araújo, Jéssica Naiara de Medeiros Araújo, Ana Beatriz Marinho de Medeiros, Pedro Henrique Azevedo dos Santos, Bárbara Ebilizarda Coutinho Borges, Jéssica Dantas de Sá Tinôco, Héllyda de Souza Bezerra

**Affiliations:** 1 Universidade Federal do Rio Grande do Norte, Natal, RN, Brasil.; 2 Universidade Federal do Rio Grande do Norte, Santa Cruz, RN, Brasil.; 3 Universidade do Estado do Rio Grande do Norte, Caicó, RN, Brasil.

**Keywords:** Prática Avançada de Enfermagem, Pesquisa em Enfermagem, Programas de Pós-Graduação em Saúde, Indicadores de Produção Científica, Bibliometria

## Abstract

**Objetivo:**

Analisar a produção científica sobre a Prática Avançada de Enfermagem a partir das dissertações e teses publicadas no Brasil.

**Método:**

Estudo bibliométrico, com abordagem descritiva de base documental, realizado no Banco de Teses e Dissertações da Coordenação de Aperfeiçoamento de Pessoal de Nível Superior, *Scientific Electronic Library Online*, Biblioteca Virtual em Saúde, Literatura Latino-Americana e do Caribe em Ciências da Saúde e repositórios institucionais, no período de outubro a novembro de 2023.

**Resultados:**

Das 25 produções científicas identificadas, 16 são dissertações e nove são teses. A maioria foi publicada em 2022 (40,0%), na região Sudeste (60,0%), especialmente na Faculdade Israelita de Ciências da Saúde Albert Einstein, pelo programa de mestrado profissional em enfermagem (20,0%). A saúde da família foi a área de especialidade mais evidenciada na amostra (36,0%), e o nível de complexidade prevalente foi a Atenção Primária à Saúde (76,0%).

**Conclusão:**

O cenário das produções científicas sobre a temática no país começa a avançar nos últimos anos, mas ainda é incipiente. O mapeamento de teses e dissertações sobre a Prática Avançada de Enfermagem aponta para a emergente necessidade de atender às demandas advindas do setor de saúde a nível internacional e nacional.

## INTRODUÇÃO

A Organização Mundial da Saúde (OMS) constantemente debate sobre medidas para atender às demandas de saúde, superando desafios como a escassez de recursos humanos e disparidades no acesso à saúde. Levando em consideração as necessidades de saúde das populações, é primordial ter modelos de saúde com potencial para inovar e aprimorar os serviços de saúde(1). Em países como Canadá e Estados Unidos, a Prática Avançada de Enfermagem (PAE) desempenha um papel relevante ao impulsionar inovações nos sistemas de saúde, melhorar o acesso aos serviços de saúde e alcançar resultados de saúde adequados, ao mesmo tempo que contribui para a redução dos custos(2,3).

De acordo com os dados do relatório *State of the world’s nursing 2020*, a existência da PAE é de 55% entre os países da região das Américas, sendo mais frequente em países com menor densidade de profissionais médicos, o que contribui para ampliar o acesso aos cuidados de saúde primários, especialmente em áreas rurais e para populações em situação de vulnerabilidade(1). No Brasil, as disparidades regionais são evidentes, com melhores indicadores de saúde no Sudeste e no Sul e piores no Norte e no Nordeste. Nesse cenário, a PAE pode ser uma estratégia a ser considerada para ampliar o acesso e melhorar a resolutividade dos serviços de saúde, alinhada aos princípios e diretrizes do Sistema Único de Saúde (SUS), sustentada por marcos legislativos, como Política Nacional de Atenção Básica, Lei do Exercício Profissional, regulamentações emitidas pelo conselho de classe, além da ênfase na prática baseada em evidências(4).

De acordo com o *International Council of Nurses* (ICN), a PAE envolve intervenções de enfermagem avançadas que impactam diretamente os resultados de saúde de indivíduos, famílias e comunidades. A PAE é fundamentada em uma formação de nível de pós-graduação, e exige a definição de critérios específicos para certificação, registro e desenvolvimento de competências essenciais para atuação(3). Uma análise conceitual definiu a PAE como “conhecimento especializado utilizado por um enfermeiro profissional licenciado e qualificado para tomar decisões complexas e avançadas e pôr em prática as habilidades clínicas necessárias para a implementação do papel da Prática Avançada na assistência à saúde, integrando teoria, prática, ensino, pesquisa, liderança e gestão”(5).

A Organização Pan-Americana da Saúde (OPAS)/OMS define a enfermeira de prática avançada (EPA) como uma profissional com formação em nível de pós-graduação que, atuando de forma integrada em equipes interprofissionais, contribui para o gerenciamento do cuidado de pacientes com doenças agudas leves e condições crônicas diagnosticadas, conforme as diretrizes ou protocolos clínicos(6).

Apesar dos avanços tecnológicos em saúde, estudiosos apontam que os sistemas de saúde não estão preparados para gerenciar as demandas de saúde dos próximos anos. Dados da OMS apontam um déficit global de 5,9 milhões de enfermeiros, com as maiores lacunas encontradas em países da África, Sudeste Asiático e em algumas partes da América Latina(1). No início de 2023, o ICN publicou o relatório “*Recover to rebuild: investing in the nursing workforce for health system effectiveness”*, no qual aponta que a escassez de enfermeiros deve ser abordada como uma emergência de saúde a nível mundial(7).

Além de formar novos enfermeiros, é imprescindível investir na formação em práticas avançadas para os enfermeiros que já estão atuando(8). No entanto, a implementação da PAE ainda está distante de se tornar realidade no Brasil. Para que seja possível avançar nessa implementação, é crucial fornecer aos enfermeiros oportunidades de desenvolvimento profissional, promover o acesso à educação formal do enfermeiro no nível de pós-graduação, implementar políticas que aprimorem as perspectivas de carreira e de mercado de trabalho, além de considerar ações para sensibilização da PAE por parte da sociedade civil, sendo imprescindível o envolvimento do governo, do setor educacional e das instituições reguladoras(9). Reforçando a relevância da temática na atualidade do Brasil, o Conselho Federal de Enfermagem (COFEN) divulgou no dia 07 de junho 2023 a Nota Técnica nº 001/2023, que aborda conceitos, ações, implementação e regulação das PAE no Brasil(10).

Nesse sentido, a análise da produção científica em PAE, a partir de teses e dissertações publicadas por enfermeiros no Brasil, justifica-se a partir da necessidade de compreender o estado atual e as tendências desse campo de atuação. Além disso, esta análise desperta a reflexão sobre a construção de conhecimento e da prática da enfermagem voltada para a temática, que pode direcionar esforços futuros, com vistas a fortalecer a base teórica e prática da PAE no Brasil.

Considerando as explanações apresentadas, tem-se como pergunta de pesquisa: qual o panorama da produção científica das dissertações e teses publicadas acerca da PAE no Brasil? Desse modo, este estudo tem como objetivo analisar a produção científica sobre a PAE a partir das dissertações e teses publicadas no Brasil.

## MÉTODO

### Desenho do Estudo

Trata-se de estudo bibliométrico, com abordagem descritiva de base documental. A bibliometria é uma abordagem de pesquisa que envolve a quantificação da produção e da comunicação científica, com o propósito de disseminar trabalhos, divulgar publicações, avaliar a produtividade de autores e instituições, revelando o desenvolvimento da ciência e o impacto das publicações no contexto estudado(11).

Desse modo, este estudo configura-se como uma pesquisa bibliográfica, utilizando materiais previamente publicados sobre a temática de PAE. Conforme Marconi e Lakatos (2017), a pesquisa bibliográfica não se limita a reproduzir o que já foi divulgado, mas busca oferecer uma análise sob uma nova perspectiva, com o intuito de alcançar conclusões inovadoras e orientar ações futuras(12).

### Protocolo do Estudo

Foi estruturado previamente um protocolo para orientar o desenvolvimento do estudo. O relatório da pesquisa foi guiado pelo *checklist Preferred Reporting Items for Bibliometric Analysis* (PRIBA), que é um instrumento utilizado para avaliação dos itens necessários para estudos bibliométricos(13).

### Coleta de Dados

O descritor “Prática Avançada de Enfermagem”, indexado nos Descritores em Ciências da Saúde (DeCS), foi selecionado como foco principal da pesquisa, por tratar-se do objeto de estudo. Vale ressaltar que um teste piloto foi realizado, utilizando o descritor selecionado, para visualizar como a pesquisa se apresentava em cada mecanismo de busca e identificar possíveis palavras-chave sobre a temática.

Após esse processo, observaram-se nos estudos palavras-chave como “Prática Avançada em Enfermagem”, “Enfermagem de Prática Avançada” e “Enfermagem em Prática Avançada”. Dessa forma, além do descritor indexado, optou-se por considerar as palavras-chave identificadas, a fim de ampliar a busca pelas produções científicas.

Para seleção dos estudos, uma busca foi realizada por dois pesquisadores no período de outubro a novembro de 2023, através do Banco de Teses e Dissertações da Coordenação de Aperfeiçoamento de Pessoal de Nível Superior (CAPES) e nas bases de dados *Scientific Electronic Library Online* (SciELO), Biblioteca Virtual em Saúde (BVS) e Literatura Latino-Americana e do Caribe em Ciências da Saúde (LILACS), atendendo às suas particularidades, com a seguinte estratégia: “Prática Avançada de Enfermagem” *OR* “Prática Avançada em Enfermagem” *OR* “Enfermagem de Prática Avançada” *OR* “Enfermagem em Prática Avançada”.

Os repositórios institucionais dos programas de pós-graduação do Brasil também foram consultados. Nestes, utilizaram-se o descritor e as palavras-chave de forma individual, sem o uso de operadores booleanos. Os repositórios institucionais foram identificados pelo acesso na Plataforma Sucupira, na aba de Cursos Avaliados e Reconhecidos, com o filtro de “Enfermagem” no item de Área de Avaliação(14). Dessa forma, foi possível emitir uma lista de 61 instituições de ensino superior (IES) com os 85 programas de pós-graduação reconhecidos na área da enfermagem no Brasil.

### Critérios de Seleção

Incluíram-se estudos que abordassem a temática e que se enquadraram como teses ou dissertações e publicados no Brasil. Não foi estabelecido um recorte temporal, a fim de ampliar a seleção dos estudos. Os estudos duplicados foram removidos da amostra.

### Análise e Tratamento dos Dados

Foi elaborado um instrumento para extração das informações contidas nas produções, com variáveis relacionadas aos dados de identificação como: título, autor, orientador, coorientador, titulação, tipo de produção, ano de publicação, instituição, programa de pós-graduação e área de concentração do programa de pós-graduação, financiamento (código) e agência de fomento; aspectos metodológicos, incluindo objetivo, questão de investigação, referencial teórico e/ou metodológico e/ou filosófico, metodologia, área de especialidade (campo de conhecimento específico da enfermagem abordado na pesquisa) e nível de atenção à saúde (nível primário, secundário ou terciário, referente ao nível de complexidade dos serviços de saúde); e principais achados, com resultados e/ou impressões dos estudos e limitações encontradas.

Nas variáveis relacionadas aos achados (resultados e/ou impressões dos estudos e limitações encontradas), foi realizada a análise temática com abordagem indutiva e baseada nos dados compilados. Após a coleta dos dados, procedeu-se a uma leitura exaustiva e detalhada, permitindo uma imersão completa no conteúdo dos estudos. Os dados foram agrupados para formar temas potenciais que, posteriormente, foram refinados para capturar as nuances presentes nos textos analisados(15). Todas as variáveis foram analisadas por meio de estatística descritiva simples, registrando-se as frequências absolutas e relativas.

### Aspectos Éticos

Como a pesquisa utilizou dados secundários de domínio público disponíveis na literatura, não foi necessária a aprovação por Comitê de Ética em Pesquisa (CEP). Contudo, cabe ressaltar que os direitos autorais dos estudos analisados foram respeitados adequadamente.

### Disponibilidade de Dados

O conjunto de dados deste estudo foi disponibilizado no repositório público *Harvard Dataverse*(16).

https://doi.org/10.7910/DVN/1N7KSG

## RESULTADOS

Foram identificadas 2.779 produções científicas, das quais 25 permaneceram na amostra. Destas, 16 são dissertações e nove são teses (Figura 1).

**Figura 1:** Fluxograma do processo de busca e seleção da amostra. Natal, RN, Brasil, 2023.

A caracterização dos estudos analisados foi apresentada na Tabela 1 a seguir, conforme ano de publicação, região geográfica do país, instituição de ensino, área de concentração do programa de pós-graduação, referencial teórico e/ou metodológico e/ou filosófico utilizado, desenho do estudo, tipo de abordagem, área de especialidade e nível de atenção à saúde.

**Tabela 1:** Caracterização das produções científicas de teses e dissertações – Natal, RN, Brasil, 2023.

Variável	N (%)
**Ano de publicação**	
2023	05 (20,0)
2022	10 (40,0)
2021	05 (20,0)
2020	03 (12,0)
2019	01 (4,0)
2018	01 (4,0)
**Região geográfica**	
Sudeste	15 (60,0)
Sul	05 (20,0)
Nordeste	03 (12,0)
Centro-Oeste	02 (8,0)
**Instituição de ensino**	
Faculdade Israelita de Ciências da Saúde Albert Einstein	05 (20,0)
Universidade de São Paulo	04 (16,0)
Universidade Federal do Paraná	02 (8,0)
Universidade Federal do Rio Grande do Norte	02 (8,0)
Universidade Federal do Estado do Rio de Janeiro	02 (8,0)
Instituto Sírio-Libanês de Ensino e Pesquisa	01 (4,0)
Escola Nacional de Saúde Pública Sérgio Arouca da Fundação Oswaldo Cruz	01 (4,0)
Universidade Estadual de Campinas	01 (4,0)
Escola de Enfermagem Aurora de Afonso Costa da Universidade Federal Fluminense	01 (4,0)
Universidade Estadual do Oeste do Paraná	01 (4,0)
Universidade Federal de Santa Catarina	01 (4,0)
Universidade Federal de Ciências da Saúde de Porto Alegre	01 (4,0)
Universidade Federal de Sergipe	01 (4,0)
Universidade de Brasília	01 (4,0)
Universidade Católica de Brasília	01 (4,0)
Área de concentração do programa de pós-graduação	
Práticas profissionais em saúde e enfermagem	05 (20,0)
Enfermagem em saúde coletiva	03 (8,0)
Práticas profissionais em enfermagem	02 (8,0)
Enfermagem na atenção à saúde	02 (8,0)
Tecnologia e inovação em enfermagem	01 (4,0)
Cuidado e inovação tecnológica em saúde e enfermagem	01 (4,0)
Ensino na saúde e suas interfaces com o sus	01 (4,0)
Enfermagem, saúde e cuidado na sociedade	01 (4,0)
Práticas e políticas de saúde	01 (4,0)
Gestão e cuidado no contexto do sus e as políticas em saúde e enfermagem	01 (4,0)
Políticas, planejamento, gestão e cuidado em saúde	01 (4,0)
Educação e trabalho em saúde e enfermagem	01 (4,0)
Fundamentação teórica, metodológica e tecnológica do processo de cuidar	01 (4,0)
Bases fundamentais, culturais, ambientais e históricas do cuidado em saúde	01 (4,0)
Longevidade e qualidade de vida	01 (4,0)
Integração ensino e serviços de saúde na formação profissional	01 (4,0)
Pesquisa aplicada em oncologia	01 (4,0)
**Referencial teórico e/ou metodológico e/ou filosófico**	
*Teórico*	
Prática Avançada de Enfermagem da ICN	06 (24,0)
Processo de Enfermagem	04 (16,0)
Competências de Zarifian	03 (12,0)
Teoria Crítica de Horkheimer	01 (4,0)
Teoria da Complexidade de Edgar Morin	01 (4,0)
Neo-Institucionalismo	01 (4,0)
*Metodológico*	
Ciência da Implementação	01 (4,0)
*Filosófico*	
Materialismo Histórico-Dialético de Karl Marx	01 (4,0)
*Não descreveram referencial de qualquer natureza*	06 (24,0)
**Desenho do estudo**	
Estudo exploratório	10 (40,0)
Estudo metodológico	06 (24,0)
Estudo transversal	02 (8,0)
Estudo de implementação	02 (8,0)
Estudo de métodos mistos	02 (8,0)
Estudo descritivo	01 (4,0)
Estudo de reflexão	01 (4,0)
Pesquisa convergente assistencial	01 (4,0)
**Tipo de abordagem**	
Mista	14 (56,0)
Quantitativa	06 (24,0)
Qualitativa	05 (20,0)
Área de especialidade	
Saúde da família	09 (36,0)
Enfermagem oncológica	03 (12,0)
Processo de enfermagem	02 (8,0)
Saúde da criança	02 (8,0)
Saúde do idoso	02 (8,0)
Saúde coletiva	01 (4,0)
Enfermagem em urgência e emergência	01 (4,0)
Gerenciamento de ações e serviços de saúde e enfermagem	01 (4,0)
Cuidados intensivos	01 (4,0)
Enfermagem obstétrica	01 (4,0)
Saúde da mulher	01 (4,0)
Saúde mental	01 (4,0)
**Nível de atenção à saúde**	
Nível primário	19 (76,0)
Nível terciário	04 (16,0)
Nível secundário	02 (8,0)

Os resultados e/ou impressões dos estudos foram analisados e demonstraram potencialidades e barreiras para implementação da PAE, conforme apresentado na Tabela 2.

**Tabela 2:** Potencialidades e barreiras para implementação da Prática Avançada de Enfermagem – Natal, RN, Brasil, 2023.

Potencialidades para implementação da Prática Avançada de Enfermagem	N (%)
Formação para a Prática Avançada de Enfermagem	22 (44,0)
Políticas públicas da Atenção Primária à Saúde	08 (32,0)
Lei do Exercício Profissional da enfermagem	08 (32,0)
Práticas em realização consideradas como Prática Avançada de Enfermagem	04 (16,0)
**Barreiras para implementação da Prática Avançada de Enfermagem**	
Necessidade de reformulação na legislação vigente	03 (12,0)
Corporativismo profissional	03 (12,0)
Falta de clareza no papel profissional na Prática Avançada de Enfermagem	03 (12,0)
Competências de gestão do cuidado ausentes	02 (8,0)
Escassez de corpo docente específico para formação para Prática Avançada de Enfermagem	01 (4,0)

As limitações dos estudos também foram analisadas, e observou-se que a maioria estava relacionada às limitações metodológicas do desenho de estudo escolhido (44,0%). O cenário pandêmico da COVID-19, atrelado à sobrecarga dos enfermeiros nesse período, também foi caracterizado como um fator limitante (16,0%), assim como a falta de legislação específica no Brasil para o exercício de PAE (8,0%) e limitações sociais e culturais descritas durante programa de doutorado sanduíche no exterior (4,0%). Em cinco produções (20,0%), não foram descritas limitações.

O demonstrativo geral das produções científicas foi disposto de acordo com a região, título, autor, ano, tipo de produção, instituição, financiamento (código) e agência de fomento (Quadro 1).

**Quadro 1:** Demonstrativo geral das produções científicas selecionadas – Natal, RN, Brasil, 2023.

Título	Autor (ano)	Tipo de produção	Instituição	Financiamento (código)	Agência de fomento
**Sudeste**					
Consulta de enfermagem em saúde da criança e competências para enfermeiros de prática avançada	Keila Gisele Lima Reis (2023)	Dissertação de mestrado – profissional	Faculdade Israelita de Ciências da Saúde Albert Einstein	Sim (001)	CAPES/COFEN
Consulta de enfermagem à pessoa idosa na atenção primária à Saúde: análise das competências propostas para enfermeiro de prática avançada	Nayara Vilela de Farias Serranegra (2023)	Dissertação de mestrado – profissional	Faculdade Israelita de Ciências da Saúde Albert Einstein	Não informado	Não informado
Consultas de enfermagem em saúde mental na atenção primária: análise das competências para enfermeiro de prática avançada	Patrícia Aline de Almeida (2023)	Dissertação de mestrado – profissional	Faculdade Israelita de Ciências da Saúde Albert Einstein	Sim (001)	CAPES/COFEN
Eventos agudos na Atenção Primária à Saúde: uma análise das competências de prática avançada em consultas de Enfermagem	Marília Orlandelli Carrer (2023)	Dissertação de mestrado – profissional	Faculdade Israelita de Ciências da Saúde Albert Einstein	Sim (001)	CAPES/COFEN
Competências de prática avançada nas consultas de enfermagem em saúde da mulher na atenção primária: estudo multicêntrico	Carla Pereira Barreto (2023)	Dissertação de mestrado – profissional	Faculdade Israelita de Ciências da Saúde Albert Einstein	Sim (001)	CAPES/COFEN
Desenvolvimento do modelo de atuação da enfermagem de prática avançada no cuidado do paciente com câncer colorretal	Michelle Artioli Domingues (2022)	Tese de doutorado – acadêmico	Instituto Sírio-Libanês de Ensino e Pesquisa	Não informado	Não informado
Processo e relações de trabalho das Enfermeiras na Atenção Primária à Saúde: uma abordagem institucional a partir do modelo de gestão do município do Rio de Janeiro/RJ	Priscilla Oliveira da Silva (2022)	Tese de doutorado – acadêmico	Escola Nacional de Saúde Pública Sérgio Arouca da Fundação Oswaldo Cruz	Sim (001)	CAPES
Adaptação Cultural e Validação do Inventario para *la Evaluacion de Competencias En Enfermeras de Practica Avanzada* (IECEPA) para a cultura Brasileira	Flávia Carvalho Pena Dias (2022)	Dissertação de mestrado – acadêmico	Universidade Estadual de Campinas	Sim (001)	CAPES
Consulta de enfermagem de 1ª vez em quimioterapia: contribuições para a prática avançada em oncologia no atendimento ambulatorial	Rubislene Assis Santos de Brito (2022)	Dissertação de mestrado – acadêmico	Universidade Federal do Estado do Rio de Janeiro	Não informado	Não informado
Prática Avançada de Enfermagem na Estratégia Saúde da Família: necessidade de aplicativo para a comunicação entre enfermeiro e usuário	Emerson Willian Santos de Almeida (2022)	Dissertação de mestrado – acadêmico	Escola de Enfermagem de Ribeirão Preto da Universidade de São Paulo	Sim (001)	CAPES
Redução da contenção mecânica em Unidade de Terapia Intensiva: implementação de um programa de melhoria	Bruna Luísa Melo de Aquino Lemos Corrêa (2022)	Dissertação de mestrado – profissional	Escola de Enfermagem Aurora de Afonso Costa da Universidade Federal Fluminense	Sim (001)	CAPES
A Enfermagem de Prática Avançada no processo de trabalho do enfermeiro da Atenção Primária à Saúde no município do Rio de Janeiro	Antonio da Silva Ribeiro (2021)	Tese de doutorado – acadêmico	Universidade Federal do Estado do Rio de Janeiro	Não informado	Não informado
Vivências dos enfermeiros em práticas avançadas nos serviços de atendimento móvel de urgências	Giane Alves Stefani (2020)	Dissertação de mestrado – profissional	Escola de Enfermagem de Ribeirão Preto da Universidade de São Paulo	Não informado	Não informado
Práticas avançadas de enfermagem na atenção primária à saúde: subsídios para o desenvolvimento e a implementação em um sistema local de saúde	Manoel Vieira de Miranda Neto (2019)	Tese de doutorado – acadêmico	Escola de Enfermagem da Universidade de São Paulo	Não informado	Não informado
Competências para práticas avançadas de enfermagem na atenção primária à saúde no contexto brasileiro	Talita Rewa (2018)	Dissertação de mestrado – acadêmico	Escola de Enfermagem da Universidade de São Paulo	Não informado	Não informado
**Sul**					
Marcadores da prática avançada de enfermagem em saúde da criança na atenção primária à saúde	Ana Paula Dezoti (2022)	Tese de doutorado – acadêmico	Universidade Federal do Paraná	Não informado	Não informado
Validação e adaptação transcultural do instrumento *Modified Advanced practice nursing role delineation tool* para o Português do Brasil	Kamila Caroline Minosso (2022)	Dissertação de mestrado – acadêmico	Universidade Estadual do Oeste do Paraná	Não informado	Não informado
Enfermagem de prática avançada em oncologia: proposta de formação profissional	Franciane Schneider (2021)	Tese de doutorado – acadêmico	Universidade Federal de Santa Catarina	Sim (001)	CAPES
Competências do enfermeiro em práticas avançadas de Enfermagem na atenção primária à saúde	Luis Fernando Gualdezi (2021)	Dissertação de mestrado – acadêmico	Universidade Federal do Paraná	Sim (001)	CAPES
Enfermagem de Práticas Avançadas: Matriz Temática para o Desenvolvimento de Protocolos na Atenção Primária em Saúde	Ernanda Mezaroba (2020)	Dissertação de mestrado – profissional	Universidade Federal de Ciências da Saúde de Porto Alegre	Não informado	Não informado
**Nordeste**					
Práticas de enfermagem no contexto da Atenção Primária à Saúde (APS) em Sergipe	Yandra Dirce Nascimento de Castro (2022)	Dissertação de mestrado – acadêmico	Universidade Federal de Sergipe	Não informado	Não informado
Prática avançada de enfermagem: reflexões para subsidiar a implementação na atenção primária a saúde brasileira	Marjorie Dantas Medeiros Melo (2021)	Tese de doutorado – acadêmico	Universidade Federal do Rio Grande do Norte	Sim (001)	CAPES
Prática avançada de enfermagem norte-americana: subsídios para reflexões da implementação no contexto obstétrico brasileiro	Isadora Costa Andriola (2020)	Tese de doutorado – acadêmico	Universidade Federal do Rio Grande do Norte	Sim (001)	CAPES
**Centro-Oeste**					
Prática de enfermagem na Atenção Primária à Saúde no estado da Paraíba: teoria, crítica, abordagens e correlações com a *Advance Nurse Practice* (ANP)	José da Paz Oliveira Alvarenga (2022)	Tese de doutorado – acadêmico	Universidade de Brasília	Sim (não especificado)	COFEN
Grau de satisfação dos usuários idosos com o modelo internacional de atuação do enfermeiro	Isis Michelle Pereira de Castro (2021)	Dissertação de mestrado – acadêmico	Universidade Católica de Brasília	Não informado	Não informado

A distribuição das produções científicas de acordo com a região brasileira, tipo de produção e área de especialidade foi apresentada na Figura 2.

**Figura 2:** Distribuição das produções científicas de acordo com a região brasileira, tipo de produção e área de especialidade. Natal, RN, Brasil, 2023.

## DISCUSSÃO

A maioria das produções científicas foi desenvolvida por mestres, com ênfase na formação acadêmica. Este achado pode estar associado ao quantitativo de cursos de mestrado e doutorado. No cenário atual no Brasil, há um maior número de programas de mestrado em comparação aos de doutorado, e os cursos voltados para a formação acadêmica são evidenciadas em relação aos de formação profissional(17).

Identificou-se que todas as produções foram desenvolvidas nos últimos cinco anos. Desde 2013, a OPAS/OMS tem impulsionado a iniciativa de EPA ao disseminar documentos para abordar os recursos humanos limitados para o acesso universal em saúde. A OPAS/OMS tem incentivado os países da América Latina e do Caribe a desenvolver e implementar a EPA nos sistemas de saúde, sobretudo no contexto da APS(18). Em 2018, a OPAS/OMS lançou o documento “*Expanding the roles of nurses in primary health care*”, com destaque para a importância da enfermagem para melhorar o acesso aos serviços de saúde, ressaltando as estratégias necessárias para o desenvolvimento, implementação e avaliação da EPA(6).

A temática da PAE foi discutida por órgãos nacionais inicialmente em 2015. Sob a presidência da Prof^a^. Dr^a^. Angela Maria Alvarez (2013–2016), a Associação Brasileira de Enfermagem (ABEn) participou ativamente de discussões sobre a PAE na América Latina em eventos nos Estados Unidos e Canadá. Além disso, a ABEn apresentou contribuições na XII Conferência de Educação em Enfermagem com o tema “Práticas Avançadas na Enfermagem na Região Íbero-América”, e coordenou um grupo de trabalho com a CAPES para desenvolver diretrizes para a PAE na APS no Brasil(18).

Ainda em 2015, a ABEn e o COFEN participaram de uma reunião na sede da OPAS, com o intuito de revisar o conceito de EPA, discutir sua proposta no Brasil e avaliar as contribuições da enfermagem na APS no âmbito das políticas públicas. Esse esforço resultou no documento “A formação em enfermagem de prática avançada na atenção primária em saúde: proposta para o Brasil”, que sugeria a ampliação do escopo da enfermagem voltada para a APS e o fortalecimento do SUS(18). Em 2016, o COFEN estabeleceu a Comissão de Práticas Avançadas em Enfermagem, com o intuito de expandir o escopo profissional e fomentar a colaboração multiprofissional na APS(19). A partir disso, observou-se o empenho no desenvolvimento de produções científicas sobre a PAE.

Notou-se também um aumento de estudos sobre a temática publicados em periódicos nacionais nos últimos anos, o que corrobora o aumento de produções científicas no mesmo período identificado no nosso estudo^(20–22)^.

Os achados do nosso estudo apontam a região Sudeste com maior número de produções sobre a PAE. Em contrapartida, não foi identificada nenhuma produção na região Norte. Acredita-se que este achado esteja relacionado à quantidade discrepante de programas de pós-graduação entre as regiões do país. A região Norte possui apenas quatro programas (dois mestrados acadêmicos e dois mestrados profissionais), número consideravelmente inferior ao das regiões Sudeste (30 programas), Nordeste (21 programas), Sul (17 programas) e Centro-Oeste (sete programas)(14). Este resultado sugere a necessidade de incentivos que possibilitem a contribuição dessa região para o avanço da ciência, por meio da instauração de novos programas e do desenvolvimento de pesquisas sob o ponto de vista regional, uma vez que o Brasil é um país caracterizado por uma alta diversidade cultural e de saúde, e cada região possui particularidades e especificidades, considerando-se, assim, a relevância da participação da região Norte(23).

A maior parte das produções foi desenvolvida na Faculdade Israelita de Ciências da Saúde Albert Einstein. Este destaque pode estar relacionado à área de concentração do programa, “práticas profissionais em saúde e enfermagem”, ao financiamento vinculado às pesquisas e ao orientador. Todas as pesquisas da instituição receberam apoio do Edital Nº 28/2019 - CAPES/COFEN, que concedia recursos de custeio aos cursos de mestrado profissional em enfermagem em IES. O orientador responsável por todos os trabalhos, Prof. Dr. Manoel Vieira de Miranda Neto, tem como principais áreas de pesquisa a APS, PAE, recursos humanos em saúde e educação interprofissional(24).

O estudo exploratório foi a metodologia mais considerada para o desenvolvimento das produções. Acredita-se que esteja relacionado aos objetivos das pesquisas que, em sua maioria, buscavam identificar as competências dos profissionais enfermeiros na PAE. Estudos apontam a urgência de identificar essas competências no contexto brasileiro para que se alcance a implementação da prática avançada(10,25).

Quando consideramos o nível de atenção à saúde em que as pesquisas foram desenvolvidas, a APS foi o nível mais comum na amostra. Consequentemente, a área de especialidade com maior número de produções foi a área da saúde da família. Este resultado está em consonância com demais pesquisas, já que as ações de enfermagem na APS no Brasil se assemelham mais às práticas avançadas(20). Estudo aborda um modelo eficaz para apoiar o processo de desenvolvimento e implementação da PAE na APS, indicando possíveis contribuições para fortalecer a enfermagem enquanto categoria profissional e seu protagonismo no sistema de saúde nacional(26).

Entre as potencialidades para a PAE no contexto brasileiro, destaca-se a proposta de que a associação da residência profissional em saúde com o mestrado profissional representa uma possibilidade promissora para a formação para a PAE. Em 2021, a CAPES formou um grupo de trabalho para discutir a formação de EPA no Brasil. Em 2022, cinco propostas de formação em EPA foram submetidas à CAPES. No entanto, em 2023, o resultado apontou que nenhuma das propostas foi aprovada, com o argumento de que a PAE não está regulamentada no Brasil. Pesquisadoras da área discordam dessa decisão, com o argumento de que a falta de regulamentação, que está em discussão, não deveria impedir a criação de novos processos de formação(27,28).

Diversos elementos podem contribuir para a definição do escopo profissional e regulamentação, incluindo a Lei do Exercício Profissional, a Política Nacional da Atenção Básica e as linhas de cuidado reconhecidas pelo Ministério da Saúde. No âmbito da APS, a PAE tem o potencial de ir além do modelo biomédico, curativo e intervencionista, ao incorporar uma abordagem mais abrangente e integradora, como a clínica ampliada(29).

Já existem atividades consideradas como PAE no cenário brasileiro, incluindo classificação de riscos com avaliações avançadas de saúde, regulação com referência e contrarreferência, tomada de decisão sobre prescrição de medicamentos e tratamentos, e solicitação de exames laboratoriais e exames de imagens, como ultrassonografia(10,30). Algumas atividades de prática avançada, como inserção de dispositivo intrauterino, também pode ser realizada por enfermeiros, embora necessite de protocolos municipais(31).

Em relação às barreiras da PAE no contexto brasileiro, as produções pontuam a necessidade de reformulação na legislação vigente, de forma a assegurar a autonomia dos profissionais na tomada de decisão. Há preocupações sobre como o país irá desenvolver a formação dos enfermeiros nessa prática, especialmente considerando a escassez de corpo docente específico. Além disso, barreiras como o corporativismo das profissões de saúde e falta de clareza no papel do profissional do enfermeiro na PAE foram identificadas na implementação da prática avançada. As pesquisas enfatizam a importância de realizar estudos colaborativos envolvendo órgãos reguladores, gestores, coordenadores, profissionais e usuários, a fim de definir claramente o perfil da PAE(32,33).

Quanto às limitações identificadas nos estudos, as limitações metodológicas são consideradas esperadas, já que todo desenho de estudo científico apresenta suas limitações inerentes. No entanto, é importante observar que o contexto da pandemia de COVID-19 e a sobrecarga enfrentada pelos enfermeiros durante esse período podem ter impactado tanto a condução das pesquisas quanto também o progresso na implementação das legislações específicas para a PAE(34,35).

Compreende-se que a PAE no Brasil se encontra em processo de implementação, devido à necessidade de mais estudos sobre o contexto brasileiro, à regulamentação das políticas educacionais e assistenciais para formação de enfermeiros com competências em PAE, e à necessidade de respaldo ético alinhado com o sistema de saúde brasileiro(22). Com base nos resultados do nosso estudo, sugerimos o estímulo a pesquisas focadas em PAE, especialmente nas regiões Norte, Centro-Oeste e Nordeste do Brasil. Isso pode envolver apoio financeiro a projetos de pesquisa, mudanças curriculares e/ou implementação de programas de pós-graduação com ênfase na formação em PAE.

Como limitações deste estudo, tem-se a possível ocultação de produções que, apesar dos esforços para identificá-las em diversos bancos de dados e em repositórios institucionais, podem não ter sido identificadas. Além disso, não foi possível avaliar a qualidade dos materiais quanto à consistência interna e realizar a análise de acoplamento bibliográfico para identificar compartilhamento de referências comuns ou semelhantes no conteúdo dos materiais.

Este estudo contribui para o avanço do conhecimento da enfermagem ao apresentar as tendências na produção científica acerca da PAE no Brasil. Identificaram-se lacunas na produção de estudos em algumas regiões que poderiam oferecer uma contribuição significativa para a discussão da PAE no país. Destaca-se que preencher essas lacunas requer maiores investimentos e uma atenção dedicada ao processo de incentivo à pesquisa nesse campo específico.

Traz contribuições também ao elencar as áreas de especialidade e os diferentes níveis de atenção à saúde nos quais a produção sobre a PAE tem sido conduzida no Brasil. Isso indica as áreas que têm potencial para a implementação da prática avançada, além de sugerir contextos propícios para a realização de pesquisas futuras. Além disso, os achados indicaram que as pesquisas provenientes da pós-graduação ainda são limitadas, especialmente considerando as possibilidades de abrangência de conteúdos e abordagens metodológicas que podem contribuir para a produção científica sobre a PAE. Portanto, recomenda-se a realização de novos estudos para preencher essas lacunas.

## CONCLUSÃO

Conclui-se que a maioria das produções científicas de teses e dissertações sobre a PAE no Brasil foi publicada em 2022, principalmente na região Sudeste, com destaque para a Faculdade Israelita de Ciências da Saúde Albert Einstein. Essas produções focaram especialmente na área da saúde da família, dentro do contexto da APS. O cenário das teses e dissertações sobre a PAE no país começa a avançar nos últimos anos, mas ainda é incipiente, considerando as potencialidades que podem ser exploradas por meio de estudos adicionais. O mapeamento de teses e dissertações sobre a PAE aponta para a emergente necessidade de atender às demandas advindas do setor de saúde a nível internacional e nacional.
